# Raman spectroscopy: the gateway into tomorrow's virology

**DOI:** 10.1186/1743-422X-3-51

**Published:** 2006-06-28

**Authors:** Phelps J Lambert, Audy G Whitman, Ossie F Dyson, Shaw M Akula

**Affiliations:** 1 Department of Microbiology & Immunology, Brody School of Medicine at East Carolina University, Greenville, North Carolina, USA

## Abstract

In the molecular world, researchers act as detectives working hard to unravel the mysteries surrounding cells. One of the researchers' greatest tools in this endeavor has been Raman spectroscopy. Raman spectroscopy is a spectroscopic technique that measures the unique Raman spectra for every type of biological molecule. As such, Raman spectroscopy has the potential to provide scientists with a library of spectra that can be used to unravel the makeup of an unknown molecule. However, this technique is limited in that it is not able to manipulate particular structures without disturbing their unique environment. Recently, a novel technology that combines Raman spectroscopy with optical tweezers, termed Raman tweezers, evades this problem due to its ability to manipulate a sample without physical contact. As such, Raman tweezers has the potential to become an incredibly effective diagnostic tool for differentially distinguishing tissue, and therefore holds great promise in the field of virology for distinguishing between various virally infected cells. This review provides an introduction for a virologist into the world of spectroscopy and explores many of the potential applications of Raman tweezers in virology.

## Background

In today's world of increasingly complex and refined biological analytical techniques, spectroscopy has maintained its place at the forefront. One type of spectroscopy in particular, Raman spectroscopy, has proven especially useful in providing detailed analysis of a staggering variety of biological samples. Raman spectroscopy is able to detect and analyze extremely small molecular objects with high resolution while eliminating outside interference [1].

Recently, a derivative of Raman spectroscopy, termed Raman tweezers, has allowed for an even greater degree of analytical capability. Raman tweezers use optical tweezers to suspend and manipulate a molecule without direct contact, so that the molecule's Raman spectra may be recorded while it is in its most natural state. As such, the spectra collected are more reflective of the true nature of the molecule under study and therefore of more significance. Even with today's advances, we are only beginning to scratch the surface of a technique that holds the promise of far-reaching and highly significant future applications.

One such field that stands to benefit greatly from Raman tweezers is virology. The high resolution, lack of sample preparation, and very short data collection time required make the technology ideal for use in the study of viruses and virally infected cells. However, because of the newness of the approach, this review has been written in such a manner that those unfamiliar with optical physics not become lost and lose interest in a technology that holds such incredible potential.

### A brief history on spectroscopy

Spectroscopy was born in 1801, when the British scientist William Wollaston discovered the existence of dark lines in the solar spectrum. Thirteen years later, Jospeh von Fraunhofer repeated Wollaston's work and hypothesized that the dark lines were caused by an absence of certain wavelengths of light [2]. It was not until 1859, however, when German physicist Gustav Kirchhoff was able to successfully purify substances and conclusively show that each pure substance produces a unique light spectrum, that analytical spectroscopy was born. Kirchhoff went on to develop a technique for determining the chemical composition of matter using spectroscopic analysis that he, along with Robert Bunsen, used to determine the chemical make up of the sun [3].

The end of the nineteenth and beginning of the twentieth centuries was marked by significant efforts to quantify and explain the origin of spectral phenomena. Beginning with the simplest atom, hydrogen, scientists including Johann Balmer and Johannes Rydberg developed equations to explain the atom's frequency spectrum. It was not until Niels Bohr developed his famous model in 1913 that the energy levels of the hydrogen spectrum could accurately be calculated. However, Bohr's model failed miserably when applied to other elements that had more than one electron. It took the development of quantum mechanics by Werner Heisenberg and Erwin Schrodinger in 1925 to universally explain the spectra of most elements [4].

From the discovery of unique atomic spectra developed modern spectroscopy. The three main varieties of spectroscopy in use today are absorption, emission, and scattering spectroscopy. Absorption spectroscopy, including Infrared and Ultraviolet spectroscopy, measures the wavelengths of light that a substance absorbs to give information about its structure. Emission spectroscopy, such as fluorescence and laser spectroscopy, measures the amount of light of a certain wavelength that a substance reflects. Lastly, scattering spectroscopy, to which Raman spectroscopy belongs, is similar to emission spectroscopy but detects and analyzes all of the wavelengths that a substance reflects upon excitation [5].

### Raman spectroscopy

Raman spectroscopy is named after the famous Indian physicist Sir Chandrasekhara Venkata Raman who in 1928, along with K.S. Krishnan, found that when a beam of light transverses a transparent chemical compound, a small fraction of that beam will emerge from the compound at right angles to and of a different wavelength from the original beam [6]. Raman received the Nobel Prize in 1930 for his work on this phenomenon, which has since been known as the Raman effect [6].

Normally, when a beam of light is shined through a transparent substance, the molecules of the substance that absorb those light wavelengths are excited into a partial quantum state (or higher vibrational state) and emit wavelengths of equal frequency as the incoming wavelengths such that there is no net change in energy between the light and the substance. Such light wavelengths are said to be elastically scattered in a process known as Rayleigh scattering [7]. On rare occasion (approximately 1/100,000 cases), the Raman Effect occurs and the molecule absorbing the incoming wavelength's energy emits a wavelength of a different frequency/energy. Of these rare occurrences, the most common are those in which a molecule releases a wavelength of lesser energy than the incoming wavelength, thereby absorbing some of the incoming wavelength's energy. These events are referred to as Stokes shifts [8]. The opposite effect may also occur, referred to as anti-Stokes shifts, in which a molecule releases a wavelength of higher energy than the wavelength it absorbs [6]. Anti-Stokes shifts are very rare; however, this is possible under certain circumstances wherein the absorbing molecule is in a partially elevated energy state prior to absorbing the incoming wavelength in order to emit a wavelength of even greater energy [4]. The ratio of these aberrant high to low wavelengths can be measured to give what is known as a Raman signal. The Raman signal given off by every type of molecule, by the interaction between different molecules, and by different thicknesses of molecules is unique, and as such, may be used to analyze a molecular species both qualitatively and quantitatively.

Raman spectroscopy is performed by illuminating a sample with a laser. The reflected light is collected with a lens and sent through a monochromator that typically employs holographic diffraction gratings and multiple dispersion stages to achieve a high degree of resolution of the desired wavelengths [6]. A charge-coupled device (CCD) camera or less commonly, photon-counting photomultiplier tube (PMT) then detects and measures those wavelengths, which are then compared to a library of known wavelengths of molecules in order to determine the composition of the tested substance [1]. Alternatively, a Fourier transform technique may be employed in which a Fourier transform is used to convert an interferogram produced from a sample into a highly accurate spectrum [9]. Unlike conventional methods, the Fourier transform technique may only be used in the near-infrared spectrum [9].

**Figure 1:**
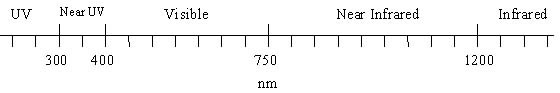
Line drawing depicting the region where Near UV and Near Infrared Wavelengths fall in the Light Spectrum.

While initial Raman spectroscopy was unable to analyze most biological samples due to the interference from the background fluorescence of water, buffers, and/or mediums present in the sample, two new types of Raman spectroscopy have been developed that solve this problem. Both types, near-infrared (NIR) and ultraviolet (UV) Raman spectroscopy, rely on using wavelengths well away from those of fluorescence. Near-infrared Raman spectroscopy relies on long near-infrared wavelengths while ultraviolet Raman spectroscopy relies on short wavelengths to avoid interference from mid-wavelength fluorescence, as shown in figure 1. UV Raman spectroscopy has a slight advantage over NIR Raman spectroscopy in better avoiding interference due to fluorescence [10].

There are four major types of Raman spectroscopy in use today: surface enhanced Raman spectroscopy (SERS), resonance Raman spectroscopy (RRS), confocal Raman microspectroscopy, and coherent anti-Stokes Raman scattering (CARS) [1]. SERS, which absorbs molecules onto a rough gold or silver surface, has the advantage of providing anywhere from a thousand to ten-million fold enhancement of the Raman signal [11,12]. In addition, the use of gold or silver in this technique removes any interference from fluorescence [13]. Unfortunately, SERS can only be used to analyze charged analytes, and therefore has only limited use in biological applications [11]. RRS also provides a marked increase in the Raman signal, but does so by taking advantage of the one hundred to one million fold signal enhancement that a molecule emits when exited at a wavelength near its transition state [14]. Unfortunately, RRS is sometimes hindered by fluorescent interference [1]. Recently, SERS and RRS have been combined to produce surface enhanced resonance Raman scattering (SERRS), a system that combines the signal enhancement of both RRS and SERS and the SERS's avoidance of fluorescence to produce ultra-sharp spectrographs. To date, SERRS has proven to be extremely useful in DNA detection [15].

The second two types of Raman spectroscopy, confocal Raman microspectroscopy and coherent anti-Stokes Raman scattering (CARS) are not only able to analyze nearly all biological samples, but also avoid any fluorescent interference. Both confocal Raman microspectroscopy and CARS spectroscopy get around this problem of fluorescence in unique ways. Confocal Raman microspectroscopy eliminates any lingering fluorescence by measuring the Raman spectra of micro regions of a sample one at a time such that the effects of fluorescence are eliminated while high resolution is maintained [16]. Because this method measures micro regions individually, it also has the advantage of being able to detect and isolate small individual biological molecules that other techniques cannot. The major disadvantage of using confocal Raman microspectroscopy, is the long time (several hours) the technique requires to produce a Raman image [17]. CARS spectroscopy eliminates the effects of fluorescence by combining the beams from two lasers to create a single high energy beam that is so strong that the Raman spectra it produces can be detected over background fluorescence [16,18]. This system also has the advantage, since it computes nonlinear (quadratic, cubic, and quartic) functions of the electromagnetic field strength, of being able to determine a molecule's chirality [19]. The largest drawback of CARS despite current work to resolve it, is its relative inability to distinguish between small equally sized molecules [16].

### Applications of Raman spectroscopy

With the issue of background fluorescence solved, Raman spectroscopic analysis has become an analytical method of choice in an extremely wide range of biological applications. Some of the more obscure applications of this technique include everything from determining the molecular structure of the skin of a 5200 year old frozen man to the analysis and authentication of foods such as olive oil and Japanese sake [20-22]. One of the more significant applications has been in pharmaceutical research and development, where Raman spectroscopy has been applied in duties ranging from shelf-life assessment and drug formula characterization to non-invasive pharmacokinetic analysis [9,23,24].

Of even greater consequence, perhaps, has been Raman spectroscopy's contribution to detailed cellular analysis. Modern techniques have allowed for the Raman spectroscopic analysis of cells *in vivo *without the need of fixatives, thereby providing extremely detailed analysis of cells in their natural state [25]. Such analytical potential has been put to good use in not only completing spectral maps but also monitoring the changes over time of numerous varieties of cells, including bacteria and many eukaryotes [25,26]. For example, Raman spectroscopy has been applied to everything from studying lipid droplet and other particulate levels in human cells to finding lignin radicals in plant cell walls and monitoring bacterial levels in drinking water [27-29]. Additionally, as is shown in table 1, the necessary steps and time required in sample preparation is much less in Raman spectroscopy than with other analytical methods.

**Table 1:** Raman tweezers is compared to other analytical techniques in terms of their time and sample processing requirements.

	**Experimental techniques**					
	
**Steps involved in processing cells**	Raman tweezers	Western blotting	Northern blotting	Southern blotting	IFA	Flow cytometry
**Washing**	-	+	+	+	+	+
**Lysing**	-	+	+	+	+	+
**Protein estimation**	-	+	-	-	-	-
**DNA/RNA estimation**	-	-	+	+	-	-
**Hybridization**	-	+	+	+	+	+
**Time**	5 min	1–2 days	2 days	2 days	3–4 h	3–4 h

Of particular interest has been the application of Raman spectroscopy in medicine. The technique's ability to provide detailed images of cells has allowed for the comparative analysis between numerous healthy tissues and their diseased states. Such analytical potential has been especially suited in the diagnosis of numerous cancers, including: intestinal, stomach, laryngeal, brain, breast, mouth, skin, and others [30-37]. Other applications of Raman spectroscopy outside of cancer have included bone quality assessment for improved estimates of the risk of fracture, corneal hydration gradient analysis, rapid identification of bacterial and fungal infection, and even antibiotic susceptibility testing [23,38-43].

Recently, Raman spectroscopy has been coupled with modern fiber optic technology to accurately measure tissue spectra in vivo without the need of biopsy. This method employs a small fiber optic probe that both has the capability to reach less assessable organs and only requires less than two seconds to collect spectra [44]. As such, it is very useful for determining the spectra of cells in their most natural state, and therefore ensures more accurate results. This method has been successfully used in the detection of atherosclerosis and cervical cancers, among other diseases [45,46]. Use of higher, near UV wavelengths has solved the initial problems this technology experienced with fluorescent interference [47].

The use of Raman spectroscopy in differential medicine is not limited to tissues and cells; it also has applications in virology. The technique has been put to good use in determining the structures and stereochemistry of both the protein and nucleic acid components of viruses, even going so far as to being able to distinguish between different types of right-handed DNA alpha-helixes [48-53]. Raman spectroscopy has also been used to help better characterize the conformational changes that occur leading to viral procapsid and capsid assembly [54-56]. As such, Raman spectroscopy holds the potential to distinguish between even the most similar viruses, thereby increasing its possible role even further in diagnostic medicine.

### Limits of Raman spectroscopy

The analytical capabilities of Raman spectroscopy are limited by its inability to manipulate, and therefore thoroughly analyze the biological molecules under study without making physical contact. This limitation has been resolved by coupling Raman spectroscopy with a technology called optical tweezers. The new method, termed Raman tweezers, uses optical tweezers to manipulate a sample without contact with it so that it remains unchanged for Raman spectroscopic analysis.

### Raman tweezers

Raman tweezers is a relatively new technology that couples Raman spectroscopy with optical tweezers to achieve previously unheard of sample control and resolution. Optical tweezers is a system that focuses a near-infrared laser on a sample to fix it in an optical trap from which it may then be maneuvered and controlled. The technique, which was first developed by Arthur Ashkin et al. in 1986, has the ability to control objects ranging in size from 5 nm to over 100 mm, whether they be atoms, viruses, bacteria, proteins, cells, or other biological molecules [57,58]. Perhaps most importantly, optical tweezers allows for the analysis of the sample without physically touching it or needing to absorb it to a surface, thereby leaving it in a less disturbed and more natural state [59]. As such, Raman tweezers has the capability to analyze a molecule from every angle and therefore provide more accurate information about identity, structure, and conformation than can Raman spectroscopy alone. Optical tweezers provides the further advantages of eliminating stray light and fluorescence as well as, in holding a molecule in place in an optical trap, allowing for the best possible excitation and collection of Raman spectra [60]. This optical trap also allows Raman tweezers to easily separate molecules for isolated study, such as their response to different conditions and/or treatments [61]. A schematic describing the set-up of a Raman tweezers is shown in Figure 1. The results of Raman tweezers are depicted in the form of a spectrograph (Raman spectrum profiles). Each peak on the spectrograph represents a particular molecule (example: DNA, amino acid, and amide) in the sample. The set of peaks on a spectrograph is different for every unique molecule, thereby allowing Raman tweezers to create spectroscopic "fingerprints" of molecules that can be used as reference in analytical studies.

The one major drawback of using Raman tweezers instead of Raman spectroscopy, however, is its inability to be used with fiber optic probes and therefore be applied to *in vivo *tissue analyses. Despite this drawback, Raman tweezers is a highly useful marriage of Raman spectroscopy and optical tweezers that further enhances Raman spectroscopy's analytical capabilities.

### Current applications of Raman tweezers

The potential of Raman tweezers is staggering. The technique holds all the promise of Raman spectroscopy, including the potential to identify almost any biological molecule and disease, and adds to it both a greater level of control and analytical capability as well as the capability of observing a sample in its natural state. As such, Raman tweezers is likely to surpass Raman spectroscopy in use for biological analysis.

To date, only a handful of biological molecules and processes, including red blood cells, lipoproteins, cell membrane components and T cell activation, have been studied with Raman tweezers [62-65]. Notably, Ramser and Enger et al. have taken advantage of Raman tweezer's ability to suspend red blood cells to study their reaction *in vivo *under different conditions [66]. Raman tweezers has also been employed in the study of disease in not only identifying pathogenic bacteria and spores but also discerning healthy from virally infected cells [62,67-69]. Thus, even though Raman tweezers cannot yet be coupled with fiber optics for human *in vivo *tissue analysis, its ability to manipulate a sample without physically coming into contact with it has allowed a degree of detailed analysis not possible with Raman spectroscopy alone.

### Future applications of Raman tweezers in virology

Raman Tweezers, while yet far having proven itself an enlightening diagnostic tool in virology, is still in its infancy. With proper nurturing, this technique has the potential to blossom into a truly brilliant and highly useful tool in the virologist's arsenal. As the resolution of Raman spectrographs increases, so will their analytical capabilities. It is likely in the not too distant future, that this technology will allow scientists to go beyond their current capability of distinguishing infected from healthy cells to being able to distinguish between differentially infected cells. Given a detailed library of spectra, a researcher could potentially even be able to characterize an unknown virus' structure, components, and lytic or latent state of infection. Furthermore, the technique's optical tweezers would allow for the study of the more temperamental cell lines, such as 293, that die more easily upon physical contact. All of these analytical capabilities would give the virologist a much clearer window to study viruses.

One could also use this technique's capabilities to not only characterize a virus, but also monitor the efficacy of antiviral treatments and determine viral load, among other applications. While all of these potential applications can be done today through alternative means, these processes must be completed separately and are time consuming. Raman tweezers greatly simplifies this process by providing a comprehensive analytical system that is both able to collect all the necessary data at once and able to do so in a very short time, thereby making it extremely cost effective. The process is so fast in fact (Table 1) that the progression of an infection or treatment could be studied in relative real time. This would serve investigators as an enormous tool with which to study viral processes as they progress, instead of just being able to study them from specific and distant time points. Such immediate and detailed analysis has potentially great applications in medicine in allowing for quick diagnosis and monitoring of virally infected patients. Through running a few drops of a patient's blood through a Raman spectrograph and reading their spectra, their care could be tailored to the state of their infection and the efficacy of the drugs to treat that infection. In addition, asymptomatic virally infected patients could be easily identified and treated before potentially harmful symptoms manifest themselves [70]. As such, Raman tweezers could prove to be one of the most effective analytical tools not only in the researchers', but also clinicians' repertoire.

## Conclusion

In conclusion, Raman tweezers is an extremely powerful analytical tool that provides biologists with a fingerprint of the agent they are studying and whose immense future applications are only now being fully understood. It is up to virologists, however, to realize the full scope and magnitude of these applications and to press for the development of this seemingly unrelated technology in virology.

## Competing interests

The author(s) declare that they have no competing interests.

## Authors' contributions

SMA conceived the idea, designed the outline, coordinated the project, and helped to draft this review. PJL, AGW, and OFD collected intellectual materials towards different sections of the review. In addition, PJL was instrumental in writing the first draft. All authors read and approved the final version of the manuscript.

**Figure 2:**
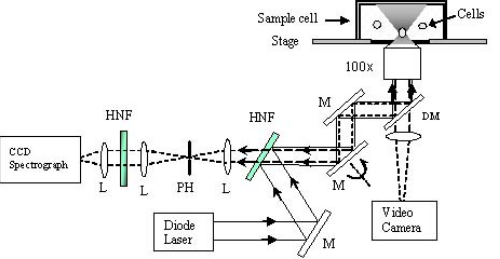
**Raman tweezers. **The figure has been adapted from Hamden et al., 2005 [67]. The figure is a schematic of a model Raman tweezers. The combined laser tweezers and Raman spectroscopy instrument possesses a laser beam at 785 nm from a wavelength-stabilized, beam shape-circulated semiconductor diode laser that is introduced into an inverted microscope through a high numerical aperture objective (100×, NA = 1.30) to form an optical trap. The same laser beam is used to excite Raman scattering of the trapped particle. The scattering light from the particle is collected by the objective and coupled into a spectrograph through a 200-μm pinhole, which enables confocal detection and rejection of off-focusing Rayleigh scattering light. A holographic notch filter is used as a dichroic beam splitter that reflects the 785-nm excitation beam and transmits the Raman shifted light. A green-filtered illumination lamp and a video camera system are used to verify trapping and observe the image of the cell. The spectrograph is equipped with a liquid-nitrogen-cooled charge-coupled detector (CCD). Abbreviations: M-mirror; L-lens; DM-dichroic mirror; PH-pinhole; HNF-holograph notch filter.
